# Dietary Acrylamide Intake and the Risk of Liver Cancer: The Japan Public Health Center-Based Prospective Study

**DOI:** 10.3390/nu12092503

**Published:** 2020-08-19

**Authors:** Ling Zha, Tomotaka Sobue, Tetsuhisa Kitamura, Yuri Kitamura, Junko Ishihara, Ayaka Kotemori, Rong Liu, Sayaka Ikeda, Norie Sawada, Motoki Iwasaki, Shoichiro Tsugane, for the JPHC Study Group

**Affiliations:** 1 Division of Environmental Medicine and Population Sciences, Department of Social and Environmental Medicine, Graduate School of Medicine, Osaka University, 2-2 Yamadaoka, Suita 565-0871, Japan; ivy_mist@outlook.com (L.Z.); lucky_unatan@yahoo.co.jp (T.K.); ytkitamura@envi.med.osaka-u.ac.jp (Y.K.); liur8939@163.com (R.L.); sayakaikeda0201@gmail.com (S.I.); 2 Department of Food and Life Science, School of Life and Environmental Science, Azabu University, 1-17-71 Fuchinobe, Chuo-ku, Sagamihara, Kanagawa 252-5201, Japan; j-ishihara@azabu-u.ac.jp (J.I.); kotemori@azabu-u.ac.jp (A.K.); 3 Epidemiology and Prevention Group, Center for Public Health Sciences, National Cancer Center, 5-1-1 Tsukiji, Chuo-ku, Tokyo 104-0045, Japan; nsawada@ncc.go.jp (N.S.); moiwasak@ncc.go.jp (M.I.); stsugane@ncc.go.jp (S.T.)

**Keywords:** acrylamide, liver cancer, diet, cohort

## Abstract

Acrylamide has been studied for its carcinogenicity in experimental animals, causing tumors at several organ sites, and has been considered probably carcinogenic to humans as well. Given the small number of epidemiological studies that have been conducted, it is still uncertain whether the consumption of acrylamide is associated with liver cancer. Therefore, we investigated a study to determine the possible relationship between acrylamide intake and the risk of developing liver cancer in the Japanese population. A total of 85,305 participants, from the Japan Public Health Center-based Prospective Study, who provided a validated food-frequency questionnaire were enrolled between 1995 and 1998. During a median of 16.0 years follow-up, 744 new liver cancer cases were identified. Compared to the lowest tertile of acrylamide consumption (<4.8 µg/day), the multivariate hazard ratio (HR) for the highest tertile (≥7.6 µg/day) was 0.79 (95% confidence interval [CI] = 0.65–0.95) for liver cancer using multivariable model 1, adjusted for smoking status, body mass index (BMI), physical activity, medical history, and alcohol consumption; whereas the inverse relationship disappeared after additionally adjusting for coffee consumption in multivariable model 2 with HR of 1.08 (95% CI = 0.87–1.34) for the highest tertile. The effect of dietary acrylamide intake on the risk of liver cancer was not observed in the Japanese population.

## 1. Introduction

Acrylamide is considered to be a potent neurotoxin agent and has been hypothesized to promote cancer risk in humans and animals, which particularly occurs via occupational exposure [1,2,3]. In 1994, the International Agency for Research on Cancer classified acrylamide as a probable human carcinogen (Group 2A) [4]. In 2002, a report from Sweden which indicated that acrylamide could be detected in carbohydrate-rich foods because of high temperature cooking over 120 degree Celsius, was aware of the concern of the harmful effects of acrylamide [5]. The formation of acrylamide depends on different cooking methods, which leads to a wide variability of acrylamide levels in the same food [6].

In western countries, although some epidemiological studies have been investigated into the effect of acrylamide consumption on the risk of cancers, the results from studies into breast cancer, endometrial cancer, rectal cancer, and lung cancer were inconsistent [7]. Currently, there are few epidemiological studies being conducted to determine the relationship between acrylamide intake from daily diet and the risk of human cancers in Asian countries. One of the reasons for this is that an appropriate method of estimating acrylamide intake has not been well-established yet. Furthermore, to our knowledge, the relationship between acrylamide consumption and liver cancer risk has not yet been investigated anywhere in the world.

The underlying mechanism of promoting the effect of acrylamide on cancers in human beings is still obscure. Although sufficient evidence from both animal experiments and epidemiological studies in humans is lacking, it has been reported that one of the pathways for acrylamide metabolism involves the process of acrylamide being converted to glycidamide, a genotoxic and mutagenic substance. This conversion occurs in the liver and is mediated by cytochrome P450 2E1 (CYP2E1) [8,9,10]. In consequence, CYP2E1 has been found to catalyze the bioactivation of some protoxins and procarcinogens, including *N*-nitrosodimethylamine. Exposure to *N*-nitrosodimethylamine has been known to develop tumors in the lung, kidney, and liver in animals [11,12]. Therefore, it is important to demonstrate the effect of acrylamide on the human liver. We conducted this study to investigate whether increasing the dietary intake of acrylamide is related to the risk of developing liver cancer in the Japanese population. To the best of our knowledge, our study is the first epidemiological study focusing on this issue.

## 2. Materials and Methods

### 2.1. Study Design and Population

The Japan Public Health Center-based Prospective Study (JPHC Study) is a prospective cohort study, established with the primary aim of establishing evidence to benefit health maintenance and improvement, including cancer prevention. Between 1990 and 1993 140,420 middle-aged Japanese adults were recruited as the target population in the JPHC Study. A self-completion questionnaire survey on lifestyle habits, including dietary habits, was conducted among the study population. Further, they were requested to provide blood samples and the results of prior local or workplace health check-ups. All cohort participants were followed up to obtain data regarding the incidence of death, migration, cancer, and cardiovascular disease. The five-year follow-up survey consisted of a second questionnaire survey among the cohort participants, being held between 1995 and 1998. We included all responders of the five-year follow-up survey, and who fulfilled the following criteria: age between 45 and 74 years old, Japanese nationality, and not registered at any age-biased designated areas [13]. A total of 93,835 men and women were enrolled into the present cohort. All subjects gave their informed consent for inclusion before they participated in the study. The study was conducted in accordance with the Declaration of Helsinki, and the protocol was approved by the Institutional Review Board of the National Cancer Center, Japan (Ethical Approval Code: 2001-021), as well as Osaka University (Ethical Approval Code: 14020-9) and Azabu University (Ethical Approval Code: 2457).

**Figure 1:**
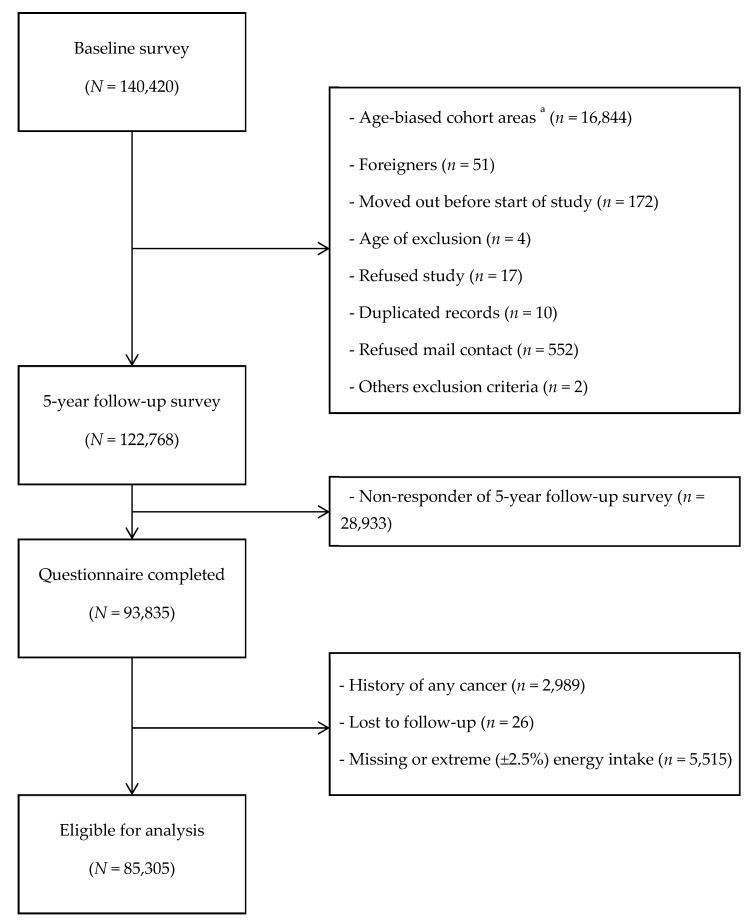
Flow diagram of eligibility for analysis. ^a^ Participants only at the age of 40 yrs. And 50 yrs. old received baseline questionnaire in these areas.

Of the initial 93,835 participants, we excluded 2989 participants who had been previously diagnosed with any cancers by the beginning of follow-up. In addition, 26 participants who were lost to follow-up were excluded from the analysis. Furthermore, we excluded 5515 participants who did not provide complete dietary data, as a result of which the total energy intake could not be calculated, and who were identified as the extreme low- and high-energy reporters. After excluding these ineligible participants, 85,305 participants were analyzed in the present study. (Figure 1)

### 2.2. Acrylamide Intake Assessment

In the JPHC Study, the detailed information on lifestyle habits, history of health status, basic social factors, and daily dietary habits were obtained through a comprehensive questionnaire, including a food frequency questionnaire (FFQ). Participants were asked to report the general average consumption frequency of 138 food and beverage items, of standard portion size, in the last year [14]. The details of response choices for frequency, standard portion size, and relative portion sizes have been described elsewhere [15,16,17]. The validity and reproducibility study of the FFQ has been conducted by taking the dietary intakes from 28-day weighed dietary records (DRs) as a reference in a subcohort of the JPHC Study [18,19,20]. Moreover, the daily nutrients intake was calculated in reference to the Standard Tables of Food Composition in Japan (5th revised and enlarged edition) [21].

The acrylamide intake assessment from daily diet was estimated by using the measured values of acrylamide content in the following common Japanese food and beverage items as follows: baked fish paste, bread, rice cakes, Japanese-style confectionary, cakes, biscuits and cookies, chocolate, peanuts, fried tofu, miso, beer, green tea, oolong tea, black tea, coffee, and soup [22,23,24,25,26,27,28]. Furthermore, we estimated the acrylamide content by considering the different cooking methods involved. The intake of acrylamide from heated starchy vegetables (potato and sweet potato), vegetables (onion, bean sprouts, sweet pepper, squash, cabbage, snap beans, and broccoli), toast, boiled or stir-fried rice, and fried batter was calculated by multiplying the amount of raw food by the proportion of heated food (as calculated from the DRs) and the concentration of acrylamide in each heated food. The validity and reproducibility of the acrylamide intake assessment were evaluated, and showed Spearman’s correlation coefficients of 0.34–0.47 reported in detail elsewhere [29].

### 2.3. Statistical Analysis

Participants contributed person-years from the five-year follow-up survey until the date of diagnosis of liver cancer, death (from any cause), moving out of study areas, or 31 December 2013, whichever occurred first. Acrylamide intake was included in the multivariable-adjusted models as a categorical variable as well as a continuous variable (per 10 µg/day), to investigate the dose-response relationship. For acrylamide intake to be modeled as a categorical variable, participants were divided into tertiles according to their energy-adjusted intakes of acrylamide, which was computed using the residual method. The groups with the highest and lowest consumption were indicated as T3 and T1, respectively. Patient characteristics at the five-year follow-up survey were compared between groups, using the Kruskal−Wallis test or Chi-square test, whichever was appropriate. A Cox proportional hazards regression model was used to estimate hazard ratios (HRs) and their corresponding 95% confidence intervals (CIs), with T1 as the reference group.

In order to assess potential confounding, besides sex, 5-year age group, and public health center area, the following variables were tested, based on the previous studies: smoking status (never, former, current, or missing), body mass index (14 to <19, 19 to <21, 21 to <23, 23 to <25, 25 to <27, 27 to <30, and 30 to 40 kg/m^2^, or missing), physical activity (quartiles of metabolic equivalents, or missing), alcohol consumption (nondrinker, <150 or ≥150 g/week, or missing), and self-reported hepatitis and diabetes mellitus (reported at the baseline survey and five-year follow-up survey) (no or yes). The hazard ratio of acrylamide intake in the multivariable-adjusted model 1 was modified using these variables. Since the previous JPHC Study, coffee consumption has been related with a lower risk of liver cancer [30]; therefore, coffee consumption (g/day; nondrinker or quintiles) was added to the above variables in the multivariable-adjusted model 2. We additionally implemented analysis stratified by coffee consumption (nondrinker and coffee drinker).

Smoking is considered as one of major sources of acrylamide exposure, and smokers have been found to have a high-level of acrylamide-hemoglobin adducts (which is a marker of the internal dose of acrylamide) that are, on an average, three to four times higher than that seen in nonsmokers [31]. Consequently, to preclude confounding through smoking status, stratification analysis was performed according to never and ever smokers.

We also used multivariable-adjusted Cox proportional hazards regression to perform sensitivity analysis by excluding participants diagnosed with liver cancer within three years of the five-year follow-up survey (*n* = 69), and to perform another sensitivity analysis by excluding participants who reported a history of hepatitis (*n* = 1762). Furthermore, we conducted the analysis by gender to confirm whether there were any gender differences. All p-values were two-tailed. P-values less than 0.05 were considered statistically significant. We performed all the statistical analyses by using Stata/MP 14.1 (StataCorp, College Station, TX, USA).

## 3. Results

The distribution of characteristics for participants at 5-year study survey are shown in Table 1. After adjusting for total energy intake, the mean intake (±SD) of acrylamide by our study population was 6.9 ± 3.8 µg per day. The major food sources of acrylamide in Japan were coffee (27.4%), green tea (21.6%), potato (11.0%), vegetables (10.8%), and biscuits (10.6%), which were different from western countries [27,30,31]. Participants who consumed more acrylamide were, on an average, younger, had lower intake of alcohol, and less likely to have diabetes or hepatitis.

During a total of 1,267,791 person-years of follow-up (14.9 years on average), 744 cases of liver cancer were ascertained among the 85,305 eligible participants. Table 2 shows that the higher energy-adjusted acrylamide intake was associated with a significantly lower risk of liver cancer in the age- and area-adjusted model and the multivariable model 1. Subjects with a high acrylamide intake (T3) had a 21% lower liver cancer risk than those who had a low acrylamide intake (T1) (HR = 0.79; 95% CI = 0.65–0.95; P trend = 0.01) in model 1. Results were similar in sensitivity analyses. However, the statistically significant decrease disappeared after additional adjustment for coffee consumption (HR = 1.08; 95% CI = 0.87–1.34). Exclusion of cases where liver cancer was diagnosed during the first three years of follow-up did not change the results in model 2. In addition, the negative result found in model 2 did not change after considering the information on hepatitis B and C virus infection. Besides, exclusion of participants who reported a history of hepatitis did not change the results in multivariable model 3.

Similar results were observed in the stratification analysis by coffee intake. Although an increased acrylamide intake reduced the risk of liver cancer among coffee drinkers in the multivariable model 1 (HR = 0.74; 95% CI = 0.58–0.94; P trend = 0.01), the inverse association disappeared after additional adjustment in the multivariable model 2 (HR = 1.05; 95% CI = 0.80–1.38). The association between acrylamide intake and liver cancer risk was not observed in either the nondrinkers or the coffee drinkers after considering the amounts of coffee consumption. We did not observe significant differences between never or ever smokers regarding the association of acrylamide intake with the risk of liver cancer. Furthermore, no gender differences in HR were observed in the present study, shown in Table 3.

## 4. Discussion

This large-scale population-based prospective cohort study is, to our knowledge, the first epidemiological study in humans to demonstrate the association between dietary acrylamide intake and the risk of liver cancer. We observed no significant association between dietary acrylamide intake and risk of liver cancer. Although an inverse association was observed in the multivariable model 1, this association was not observed after further adjustment in the multivariable model 2. These findings indicate that while coffee drinking may lower the risk of liver cancer, the acrylamide present in coffee contributes substantially to the total dietary acrylamide intake in Japan. Because of the dichotomic role of coffee consumption, we interpreted our results based on how coffee affects the risk of liver cancer, and by distinguishing the effect of acrylamide from the effect of coffee.

Several previous studies have suggested an inverse association between coffee consumption and liver cancer, both in Japan and in other countries [30,32,33,34,35]. Several potential mechanisms have been proposed through which coffee may lower the risk for developing liver cancer. Coffee is known to contain a variety of different biologically active chemical compounds including antioxidants and diterpenes. Antioxidants, for instance caffeine and chlorogenic acids, have been indicated for preventing oxidative DNA damage, modification effect on the apoptotic response, and reversing the cell cycle checkpoint function [36,37,38,39]. Besides, diterpenes, for instance kahweol and cafestol, have been shown to have anticarcinogenic properties, and may offer a protective effect against aflatoxin B1-induced genotoxicity [40,41,42]. These studies suggest that the ingredients in coffee may play an important role in protecting against the occurrence and development of liver cancer. Thus, the observed inverse association in the multivariable model 1, which did not adjust for coffee consumption, could not indicate the actual effect of dietary acrylamide intake. Because the inverse association disappeared after additional adjustment for coffee consumption in the multivariable model 2, coffee consumption, particularly the consumption of caffeine, chlorogenic acids, kahweol, and cafestol, should be considered as the confounding factor in this study.

To minimize the confounding effect from coffee consumption, stratified analysis was conducted by comparing HRs in coffee drinkers and nondrinkers. There was no association between acrylamide intake and liver cancer in nondrinkers. In coffee drinkers, no association was observed after adjustment for the amount of coffee consumption. These findings suggest that the association of reduced risk of liver cancer with the high intake of acrylamide was not valid.

The lack of association between acrylamide intake and liver cancer risk could be partly due to the low baseline range of acrylamide intake used in this study. The mean acrylamide intake for the reference group in this study was 3.4 µg per day, and for the highest intake category was 11.1 µg per day. In contrast, in the Netherlands cohort study, the mean acrylamide intake for the reference group was 9.5 µg per day, and for the highest intake category was 40.8 µg per day [43]. However, it is important to state that the values of acrylamide intake that were assessed by the FFQ were relative values. The determination of acrylamide exposure by using hemoglobin adducts as a biomarker of internal dose is necessary for accurate comparison.

The classification of acrylamide made by the IARC in 1994 was primarily based on evidence from animal models and mechanistic considerations [4]. In animal models, the dose of acrylamide exposure was much higher than the doses humans are exposed to through daily diet [44]. Consequently, the dose of acrylamide exposure differs between experimental animals and epidemiological studies in humans, and may help to interpret the null finding in the present study.

The present study has several strengths including a large sample size, the population-base and prospective design, and the completeness of case ascertainment through linkage to the population-based registries in Japan. However, there are also some limitations that should be discussed. First, the present study has some limitations regarding acrylamide intake assessment. The FFQ consists of a finite list of foods and beverages. Therefore, it may be a less accurate estimation of dietary intake when compared to a 24-h recall method. The correlation for acrylamide intake between the FFQ and the 28-day dietary records was relatively low [45]. Even though the use of FFQ has limitations in the assessment of dietary acrylamide exposure, it is the only feasible way of assessing dietary acrylamide intake over a long period of time in a large study population. Second, the participants were not divided into more groups based on dietary acrylamide intake because of the limited number of cases. Likewise, the associations between dietary acrylamide intake and liver cancer in never smokers and in noncoffee drinkers were based on analyses with a small number of cases. Third, in the present analysis, both acrylamide intake and information on covariates had only been measured once, while, it is highly possible that the participants may change their acrylamide consumption levels during the relatively long follow-up period. Therefore, these results should be interpreted cautiously.

## 5. Conclusions

In conclusion, the results of the present study indicate that there was no association between dietary intake of acrylamide and risk of liver cancer. Further studies with biomarkers of the internal dose of acrylamide are needed to investigate the carcinogenicity of acrylamide in humans.

## Figures and Tables

**Table 1:** Characteristics of participants (*n* = 85,305) according to tertile of energy-adjusted acrylamide intake at five-year-study survey.

Characteristics						Tertile of Energy-Adjusted Acrylamide Intake									
						Tertile 1			Tertile 2			Tertile 3			*p*-Value ^c^
Number of Participants						28,435			28,435			28,435			
Men, %						57.7			44.6			38.4			
Dietary variables															
				Acrylamide intake											
				Range, μg/d		0.04	−	4.81	4.82	−	7.63	7.64	−	67.11	
				Mean and SD, ^a^ μg /d		3.4	±	1.0	6.1	±	0.8	11.1	±	3.5	<0.001
				Mean and SD, ^a^ μg·kg body weight^−1^·d^−1^		0.06	±	0.05	0.11	±	0.09	0.21	±	0.23	<0.001
	Coffee, ^a^ g/d					42.0	±	57.8	107.1	±	105.4	276.5	±	283.8	<0.001
	Green tea, ^a^ g/d					313.9	±	337.9	510.7	±	423.1	764.4	±	680.8	<0.001
	Alcohol intake, ^a^ g/d					155.1	±	241.7	92.2	±	179.2	58.5	±	134.2	<0.001
	Biscuits, ^a^ g/d					0.8	±	1.2	2.0	±	2.5	5.0	±	7.9	<0.001
	Potato, ^a^ g/d					10.2	±	9.6	17.8	±	14.8	21.5	±	24.0	<0.001
	Vegetables, ^a^ g/d					180.4	±	119.8	220.9	±	128.4	231.1	±	143.3	<0.001
	Fruit, ^a^ g/d					178.9	±	161.4	219.8	±	163.0	221.1	±	168.5	<0.001
	Meat, ^a^ g/d					58.1	±	43.5	57.1	±	36.6	56.2	±	35.6	<0.001
	Fish, ^a^ g/d					85.9	±	56.1	88.4	±	48.9	83.7	±	47.9	<0.001
	Total energy intake, ^a^ kcal/d					1997.1	±	641.5	2019.3	±	610.4	1971.3	±	610.1	<0.001
Nondietary variables															
		Age at 5-year follow-up study, ^a^ y				57.8	±	7.6	57.1	±	7.9	55.9	±	8.0	<0.001
		Body mass index, ^a b^ kg/m^2^				23.7	±	3.1	23.6	±	3.0	23.4	±	3.0	<0.001
		Smoking status, %													
			Never				57.9			64.8			64.2		<0.001
			Former				10.6			8.3			6.8		
			Current				25.1			21.1			23.3		
			Missing				6.4			5.8			5.7		
		Number of cigarettes/d, ^a b^ only for current				20.5	±	14.3	21.2	±	11.9	22.7	±	11.4	<0.001
		Physical activity (METs) ^a^				33.2	±	6.4	33.2	±	6.2	33.1	±	6.1	<0.001
		Diabetes, % yes					8.3			6.6			5.4		<0.001
		Hepatitis, % yes					2.4			2.1			1.7		<0.001

^a^ Mean ± s.d. ^b^ Number of participants missing the following: body mass index: 2163; number of cigarettes per day for current smokers: 378; physical activity: 15,446. ^c^ Kruskal−Wallis test for continuous variables and Chi-square test for categorical variables.

**Table 2:** Hazard ratios (95% confidence intervals) for liver cancer according to tertile of acrylamide intake.

	Quartile of Energy-Adjusted Acrylamide Intake								
	10 μg/d		Tertile 1 (Lowest)		Tertile 2		Tertile 3 (Highest)		
	HR	(95% CI)	HR	(95% CI)	HR	(95% CI)	HR	(95% CI)	*p* for Trend
Number of subjects		85,305		28,435		28,435		28,435	
Person-years		1,267,791		417,202		425,177		425,412	
Number of liver cancers		744		311		248		185	
Age- and area-adjusted model ^a^	0.96	(0.94–0.98)	1.00	(Reference)	0.88	(0.74–1.04)	0.73	(0.60–0.88)	<0.01
Multivariable model 1 ^b^	0.96	(0.94–0.99)	1.00	(Reference)	0.90	(0.76–1.06)	0.79	(0.65–0.95)	0.01
Multivariable model 1 (excluding cases < 3 years)	0.97	(0.94–0.99)	1.00	(Reference)	0.91	(0.76–1.10)	0.82	(0.66–1.01)	0.06
Multivariable model 2 ^c^	0.99	(0.96–1.01)	1.00	(Reference)	1.00	(0.84–1.20)	1.08	(0.87–1.34)	0.51
Multivariable model 2 (excluding cases < 3 years)	0.99	(0.96–1.01)	1.00	(Reference)	0.99	(0.81–1.21)	1.08	(0.85–1.37)	0.58
Multivariable model 3 ^d^ (excluding cases with history of hepatitis)	0.99	(0.96–1.02)	1.00	(Reference)	0.95	(0.76–1.18)	1.12	(0.87–1.45)	0.47
By smoking status									
Never smoker									
Number of subjects		53,137		16,460		18,429		18,248	
Person-years		817,862		252,425		283,953		281,484	
Number of liver cancers		335		131		109		95	
Multivariable model 1	0.98	(0.94–1.01)	1.00	(Reference)	0.86	(0.66–1.11)	0.93	(0.70–1.22)	0.54
Multivariable model 2	0.99	(0.95–1.02)	1.00	(Reference)	0.95	(0.72–1.25)	1.15	(0.85–1.56)	0.42
Ever smoker ^e^									
Number of subjects		27,083		10,150		8365		8568	
Person-years		382,550		141,189		119,153		122,209	
Number of liver cancers		352		147		128		77	
Multivariable model 1	0.95	(0.92–0.99)	1.00	(Reference)	1.06	(0.83–1.35)	0.71	(0.53–0.94)	0.03
Multivariable model 2	0.99	(0.95–1.02)	1.00	(Reference)	1.17	(0.90–1.51)	1.07	(0.77–1.51)	0.52
By coffee consumption									
Nondrinker									
Number of subjects		23,104		13,603		6048		3453	
Acrylamide intake (mean ± SD, μg/d)			3.0	±1.1	6.0	±0.8	10.8	±3.1	
Acrylamide intake (range, μg/d)			0.0	−4.8	4.8	−7.6	7.6	−67.1	
Person-years		335,958		197,392		88,407		50,159	
Number of liver cancers		266		160		68		38	
Multivariable model 1	1.01	(0.96–1.06)	1.00	(Reference)	1.00	(0.74–1.34)	1.12	(0.77–1.62)	0.63
**Drinker**									
Number of subjects		62,201		14,832		22,387		24,982	
Acrylamide intake (mean ± SD, μg/d)			3.7	±0.8	6.1	±0.8	11.1	±3.5	
Acrylamide intake (range, μg/d)			0.4	−4.8	4.8	−7.6	7.6	−62.8	
Person-years		931,833		219,810		336,770		375,253	
Number of liver cancers		478		151		180		147	
Multivariable model 1	0.96	(0.93–0.98)	1.00	(Reference)	0.86	(0.69–1.07)	0.74	(0.58–0.94)	0.01
Multivariable model 2	0.98	(0.95–1.01)	1.00	(Reference)	0.97	(0.77–1.22)	1.05	(0.80–1.38)	0.73

Abbreviations: 95% CI = 95% confidence intervals. ^a^ Age- and area-adjusted model adjusted for gender, age (5-year age intervals) and area. ^b^ Multivariable model 1 additionally adjusted for: smoking status (never, former, current, missing), intake of alcohol (nondrinker, <150, ≥150 g/week, missing), body mass index (14−<19, 19−<21, 21−<23, 23−<25, 25−<27, 27−<30, 30–40 kg/m^2^, missing), physical activity (quartiles, missing), history of diabetes (yes/no), and history of hepatitis (yes/no). ^c^ Multivariable model 2 additionally adjusted for: multivariable model 1 and coffee consumption (nondrinker, quintiles). ^d^ Multivariable model 3 additionally adjusted for: smoking status (never, former, current, missing), intake of alcohol (nondrinker, <150, ≥150 g/week, missing), body mass index (14−<19, 19−<21, 21−<23, 23−<25, 25−<27, 27−<30, 30–40 kg/m^2^, missing), physical activity (quartiles, missing), and history of diabetes (yes/no). ^e^ Ever smoker was defined as former and current smoker.

**Table 3:** Hazard ratios (95% confidence intervals) for liver cancer according to tertile of acrylamide intake by gender.

	Quartile of Energy-Adjusted Acrylamide Intake								
	10 μg/d		Tertile 1 (Lowest)		Tertile 2		Tertile 3 (Highest)		
	HR	(95% CI)	HR	(95% CI)	HR	(95% CI)	HR	(95% CI)	*p* for Trend
Men									
Number of subjects		39,996		16,417		12,669		10,910	
Person-years		569,415		231,895		181,770		155,751	
Number of liver cancers		530		237		175		118	
Age- and area-adjusted model ^a^	0.95	(0.93–0.98)	1.00	(Reference)	0.91	(0.74–1.10)	0.71	(0.57–0.89)	<0.01
Multivariable model 1 ^b^	0.96	(0.93–0.99)	1.00	(Reference)	0.94	(0.77–1.14)	0.78	(0.62–0.98)	0.04
Multivariable model 2 ^c^	0.99	(0.96–1.02)	1.00	(Reference)	1.05	(0.85–1.29)	1.15	(0.88–1.50)	0.31
Women									
Number of subjects		45,309		12,018		15,766		17,525	
Person-years		698,376		185,307		243,407		269,662	
Number of liver cancers		214		74		73		67	
Age- and area-adjusted model ^a^	0.97	(0.93–1.00)	1.00	(Reference)	0.81	(0.59–1.13)	0.78	(0.56–1.10)	0.16
Multivariable model 1 ^b^	0.97	(0.94–1.01)	1.00	(Reference)	0.76	(0.55–1.06)	0.77	(0.54–1.08)	0.14
Multivariable model 2 ^c^	0.98	(0.95–1.02)	1.00	(Reference)	0.83	(0.59–1.17)	0.92	(0.63–1.32)	0.65

Abbreviations: 95% CI = 95% confidence intervals. ^a^ Age- and area-adjusted model adjusted for age (5-year age intervals) and area. ^b^ Multivariable model 1 additionally adjusted for: smoking status (never, former, current, missing), intake of alcohol (nondrinker, <150, ≥150 g/week), body mass index (14−<19, 19−<21, 21−<23, 23−<25, 25−<27, 27−<30, 30–40 kg/m^2^, missing), physical activity (quartile, missing), history of diabetes (yes/no), and history of hepatitis (yes/no). ^c^ Multivariable model 2 additionally adjusted for: multivariable model 1 and coffee consumption (nondrinker, quintiles).
